# Evaluating extraction-free PCR for rapid detection of beta-lactam antibiotic resistance in urinary tract infection

**DOI:** 10.1016/j.plabm.2025.e00516

**Published:** 2025-12-22

**Authors:** Sadia Almas, Rob E. Carpenter, Vaibhav K. Tamrakar, Aditya Sharma, Kamalpreet Suri, Salima Karki, Katelyn Kyser, Randy Sronce, Rahul Sharma

**Affiliations:** a Department of Research, OSPRI Biopath, Tyler, TX, 75703, USA; b University of Texas at Tyler, Tyler, TX, 75799, USA; c Department of Research, RetroBiotech and Research Pvt. Ltd, Jaipur, RJ, 302017, India

**Keywords:** PCR, Antibiotic resistance, CTX-M, Extended-spectrum beta-lactamases, Urinary tract infections, Molecular diagnostics, Extraction-free

## Abstract

The rapid detection of beta-lactam antibiotic resistance is crucial for guiding effective antimicrobial therapy and controlling the spread of resistant bacterial strains. *CTX-M* Group 1 extended-spectrum beta-lactamases (ESBLs) are among the most prevalent resistance determinants in Gram-negative bacteria, particularly *Escherichia coli* and *Klebsiella pneumoniae*, which are major causes of urinary tract infections (UTIs). Conventional molecular diagnostic methods for detecting *CTX-M* genes rely on nucleic acid extraction before polymerase chain reaction (PCR) amplification. However, these processes are time-consuming, labor-intensive, and resource-intensive, limiting their accessibility in low-resource and high-throughput laboratory settings. This study evaluates Direct-to-PCR (D2P) extraction-free technology as an alternative to traditional extraction-based methods for detecting *CTX-M* Group 1 genes. A comparative analysis was conducted using reference microbial isolates and clinical urine samples, testing D2P alongside silica column- and magnetic bead-based extraction methods. Quantitative PCR results demonstrated that D2P achieved comparable sensitivity and specificity to traditional extraction methods while significantly reducing sample processing time and cost. Statistical analysis revealed no significant differences (p > 0.05) in cycle threshold (Ct) values between D2P and conventional extraction-based methods, supporting its feasibility as a rapid, cost-effective alternative. The findings suggest that D2P technology may enhance antibiotic resistance surveillance, clinical diagnostics, and infection control programs by enabling faster, extraction-free molecular detection of ESBL-producing pathogens. Further studies should assess its performance in diverse sample matrices and clinical settings.

## Highlights

• Extraction-free PCR enables rapid detection of beta-lactam resistance in UTIs.
• Direct-to-PCR (D2P) eliminates the need for labor-intensive DNA extraction.
• D2P achieves sensitivity and specificity comparable to conventional methods.
• Faster sample processing reduces diagnostic turnaround and laboratory costs.
• Study supports D2P as a scalable alternative for antimicrobial resistance detection.

## 1 Introduction

**Fig. 1:**
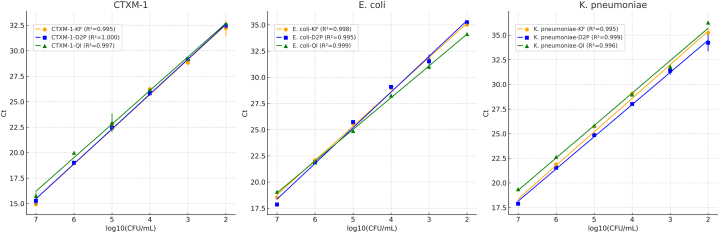
Linearity of qPCR Assays: Mean Ct values (n = 9) for CTXM-1, *E. coli*, and *K. pneumoniae* across 10^7^–10^2^ CFU/mL, comparing KingFisher (KF), Direct-to-PCR (D2P), and QIAGEN (QI) extraction workflows. Error bars show replicate variability, and regression lines demonstrate strong linearity for all methods (R^2^ ≥ 0.995).

CTX-M Group 1 is a specific subset of Cefotaximase (CTX-M) Extended-Spectrum Beta-Lactamases (ESBLs) that hydrolyze cefotaxime and other beta-lactam antibiotics, contributing to antibiotic resistance in Gram-negative bacteria [1]. They are categorized into different groups (1, 2, 8, 9, and 25) based on their amino acid sequences and phylogenetic relationships. Group 1 includes some of the most clinically significant and widely spread ESBL-producing bacteria, including CTX-M-1, CTX-M-3, CTX-M-10, CTX-M-15 and CTX-M-22. CTX-M Group 1 enzymes are critically important in infectious disease management due to their role in widespread antibiotic resistance, particularly among Enterobacteriaceae [2]. These bacteria are major causes of both hospital-acquired and community-acquired infections, making the detection and control of CTX-M-producing strains a public health priority [3]. And it foregrounds the critical importance of accurate and timely detection of CTX-M Group 1 producers in clinical settings (see Fig. 1).

The genes encoding CTX-M enzymes are often located on mobile genetic elements, such as plasmids and transposons, facilitating their rapid dissemination across diverse bacterial populations Cantón et al., 2012; [4]. This horizontal gene transfer accelerates the spread of resistance, posing notable challenges to infection control and antibiotic stewardship efforts [5]. Effective management of infections caused by CTX-M-producing organisms relies heavily on prompt and precise diagnostic methods to inform appropriate antimicrobial therapy and to implement necessary infection control measures.

Traditional molecular diagnostic methods for detecting CTX-M genes rely on nucleic acid extraction followed by polymerase chain reaction (PCR) amplification, a well-established approach that can be labor-intensive, time-consuming, and resource-intensive, potentially delaying diagnosis and treatment. To address these limitations, recent advancements have introduced extraction-free PCR techniques, which streamline the diagnostic process by eliminating the nucleic acid extraction step, offering faster turnaround times and reduced costs [6,7]. While extraction-free methods have demonstrated effectiveness in detecting viral RNA—such as SARS-CoV-2—by accelerating the diagnostic process and minimizing resource dependency, their efficacy in detecting CTX-M Group 1 genes remains underexplored. Despite their potential to preserve diagnostic accuracy while simplifying workflows, research on extraction-free technologies for CTX-M detection is still limited, highlighting a critical gap in molecular diagnostics that warrants further investigation.

This research seeks to evaluate the performance of extraction-free PCR technology in the detection of CTX-M Group 1 ESBLs. By comparing this novel approach to traditional extraction-based methods, the study aims to determine its viability as a rapid, cost-effective alternative for the identification of CTX-M-producing pathogens. The findings have the potential to impact clinical diagnostics and antibiotic stewardship programs by providing insights into more efficient detection methodologies for these critical resistance determinants.

## 2 Materials and methods

### 2.1 Study design

This study employed a comparative framework to evaluate the Direct-to-PCR (D2P) extraction-free nucleic acid processing technology (OSPRI Biopath, Tyler, TX, USA) against conventional silica column-based and magnetic bead-based extraction methods for detecting CTX-M Group 1 ESBLs. The assessment was conducted in two phases: Phase 1 benchmarked reference microbial isolates (*E. coli*, *K. pneumoniae*) while Phase 2 analyzed residual clinical specimens containing Enterobacteriaceae species harboring CTX-M Group 1 genes. In Phase 1, the OSPRI Biopath D2P method (Cat. D2P-UN-192) was compared with the QIAamp One-For-All Nucleic Acid Kit (Cat. No. 965672, QIAGEN, Hilden, Germany) and the KingFisher™ Flex Purification System (Cat. No. 5400610, Thermo Fisher Scientific, Waltham, USA). Phase 2 evaluated the D2P and KingFisher methods on residual clinical samples to assess their efficacy in isolating nucleic acids from these specimens.

### 2.2 Sample collection and preparation

#### 2.2.1 Reference microbial isolates (Phase 1)

Reference microbial isolates were obtained from the CDC Antibiotic Resistance bank selecting *Escherichia coli* harboring CTX-M-15 (CDC AR bank 591) and *Klebsiella pneumoniae harboring CTX-M-2 (*CDC AR Bank 617*)* for their clinical relevance as CTX-M Group 1 ESBL producers. Cultures were grown overnight in Luria-Bertani (LB) broth at 37 °C under aerobic conditions, then standardized to a 0.5 McFarland (∼1.5 × 10^8^ CFU/mL) after 18 h to ensure uniform bacterial density. The standardized cultures were subsequently spiked into sterile urine matrices to simulate urinary tract infection (UTI) specimens, providing a biologically relevant model for molecular detection evaluation.

#### 2.2.2 Comparative linearity and limit of detection (LOD) analysis

Linearity was evaluated using a series of 10-fold serial dilutions prepared from quantified cultures of CTXM producing *Escherichia coli* and *Klebsiella pneumoniae*. Overnight cultures were grown in Luria–Bertani broth at 37 °C, standardized to a 0.5 McFarland equivalent, and diluted in sterile urine to generate triplicate serial dilutions of 10^7^ to 10^2^ CFU/mL. Each dilution was prepared in sufficient volume for three technical replicates (n = 9) per extraction method (KingFisher, QIAGEN column, Direct-to-PCR). Aliquots were vortex-mixed, kept on ice during processing, and immediately used for extraction or direct-input workflows to minimize variability. These dilutions were used for assessing Ct–log_10_ linearity and calculating regression slopes and R^2^ values.

LOD determination was performed using fresh low-copy-number dilutions spanning 1000, 100, 10, and 1 CFU/mL, generated from the same standardized culture stocks. Each dilution was prepared immediately prior to testing to avoid freeze–thaw degradation and ensure stable low-template inputs. Three independent dilutions were tested in 8 replicates (n = 24) and processed in parallel across all three extraction workflows. Detection rates at each dilution were recorded to establish the LOD (defined as ≥95 % positivity), and the same replicate-level results were used for probit regression modeling to estimate the statistical LOD_95_.

#### 2.2.3 Residual clinical specimens (Phase 2)

De-identified residual clinical urine samples (*n* = 40) from patients suspected of harboring CTX-M-producing Enterobacteriaceae were obtained from Advanta Genetics LLC (Tyler, Texas, USA). Samples were tested for *Escherichia coli*, *Klebsiella pneumoniae*, *Enterobacter cloacae*, *Klebsiella oxytoca*, *Klebsiella aerogenes*, *Citrobacter* spp., *Proteus mirabilis*, *Proteus vulgaris*, *Morganella morganii*, and *Serratia marcescens*. All specimens were processed in accordance with institutional ethics guidelines, and the study was IRB-exempt due to the use of de-identified samples**.**

### 2.3 Nucleic acid extraction methods

Three different extraction methods were evaluated for DNA isolation from CTX-M Group 1 bacteria. These included the silica column-based extraction and magnetic bead-based extraction, and extraction-free D2P processing.

For the silica column-based extraction, genomic DNA was extracted using the QIAamp One-For-All Nucleic Acid Kit (Qiagen, Hilden, Germany). Samples were first subjected to proteinase K digestion, followed by lysis with AL buffer, after which nucleic acids were bound to silica membranes within spin columns. To remove contaminants, bound DNA underwent multiple washing steps using AW1 and AW2 buffers, followed by elution in 50 μL of elution buffer.

For the magnetic bead-based extraction, DNA was isolated using the MagMAX Viral/Pathogen Nucleic Acid Isolation Kit in conjunction with the KingFisher™ Flex Purification System (Thermo Fisher Scientific, Waltham, USA). Samples were incubated in a chaotropic buffer containing proteinase K to disrupt cellular membranes and release nucleic acids. The DNA was then captured onto paramagnetic beads, which were subjected to sequential washes with ethanol-based wash buffers to remove residual contaminants. After purification, bead-bound DNA was eluted into a low-salt buffer optimized for downstream applications, including PCR amplification.

For the extraction-free D2P method, a 1 mL urine sample was centrifuged at 10,000×*g* for 10 min at 4 °C, after which the supernatant was discarded to retain the bacterial pellet. The pellet was then resuspended in 30 μL of D2P-Universal Extraction Buffer (Cat. D2P-UN-192; OSPRI Biopath, Tyler, TX, USA) to facilitate bacterial lysis. The sample was vortexed thoroughly and subsequently heat-treated at 95 °C for 10 min to ensure complete cell lysis and release of genomic DNA. To optimize reagent conditions and stabilize nucleic acids, an additional 30 μL of D2P-Sample Dilution Buffer was added to the lysate. Finally, the processed lysate was briefly centrifuged at 8000×*g* for 10 s before being directly used in quantitative PCR **(**qPCR) for pathogen and CTX-M Group 1 gene detection**.**

### 2.4 Quantitative PCR for CTX-M group 1 detection

Next, qPCR was performed using the Bio-Rad CFX96 Touch Real-Time PCR Detection System (Bio-Rad Laboratories, CA, USA) to detect the presence of bla_CTX-M Group 1 genes. Pre-formulated multiplex qPCR reagents (OSPRI Biopath, Tyler, TX, USA) were used to ensure high sensitivity and specificity for CTX-M Group 1 detection. Each qPCR reaction was prepared in a final reaction volume of 10 μL, consisting of 2.5 μL of extracted DNA (from Qiagen, KingFisher, or D2P lysate), 7.5 μL of qPCR Master Mix, containing 250 nM of each primer (CTX-M Group 1-specific forward and reverse primers), and 0.125 nM of a fluorescent probe for real-time detection. The thermal cycling protocol was optimized to ensure efficient amplification of pathogen specific gene and CTX-M Group 1 sequences and consisted of an initial denaturation at 95 °C for 5 min, followed by 40 amplification cycles, each comprising denaturation at 95 °C for 5 s and annealing/extension at 60 °C for 30 s. The cycle threshold (Ct) value cutoff was set at < 35, with samples meeting this threshold classified as positive for CTX-M Group 1 ESBL genes. Samples failing to amplify or exhibiting Ct values ≥ 35 were considered negative, ensuring high specificity and reproducibility of the assay.

### 2.5 Data analysis

To evaluate the performance of the extraction methods multiple statistical analyses were conducted. The primary performance metric was the Ct value, which represents the number of cycles required for the fluorescence signal to exceed the background threshold. Lower Ct values indicate higher nucleic acid concentration and greater extraction efficiency. Ct values for each extraction method were recorded, and the mean Ct values with standard deviations (SD) were calculated across all samples. To assess the clinical relevance of each extraction method, five key diagnostic performance parameters were calculated using standard formulas to include sensitivity (%), specificity (%), Accuracy (%), positive predictive value (PPV), and negative predictive value (NPV). These metrics were computed for each extraction method to determine relative diagnostic reliability and efficacy in detecting CTX-M Group 1 ESBL genes. To determine whether there were statistically significant differences in Ct values among the three extraction methods (Qiagen**,** KingFisher**,** and D2P), paired t-tests were performed. Mean Ct values across methods were compared, and p-values <0.05 were considered statistically significant, indicating that the observed differences were unlikely to be due to chance.

## 3 Results

### 3.1 Phase 1: comparative analysis using *E. coli* and *K. pneumoniae*

This phase evaluated the efficiency of Direct-to-PCR (D2P) extraction-free processing compared to Qiagen (silica column-based) and KingFisher (magnetic bead-based) extraction methods using *E. coli* and *K. pneumoniae*, both CTX-M Group 1 ESBL producers.

#### 3.1.1 Cycle threshold (Ct) Comparisons

Analytical performance of the CTXM-1, *E*. *coli*, and *K*. *pneumoniae* qPCR assays was characterized using serial 10-fold dilutions spanning 10^7^ to 10^2^ CFU/mL. Linearity testing across high-range dilutions (10^7^–10^2^ CFU/mL; *n* = 9 per dilution) demonstrated excellent quantitative response with R^2^ ≥ 0.995 for all three extraction workflows—KingFisher magnetic bead (KF), Direct-to-PCR (D2P), and QIAGEN spin column (QI)—confirming precise log-linear Ct progression and extraction-method equivalence.

Analytical sensitivity/LOD was assessed using n = 24 replicates at 1000, 100, 10, and 1 CFU/mL. All methods detected 1000 and 100 CFU/mL in 100 % of the replicates, fulfilling the ≥95 % positivity requirement and establishing 100 CFU/mL as the empirical LOD for all workflows. Positivity rate was gradually reduced to below 80 % for lower dilutions (Table-1). Probit regression was used to model detection probability across the dilution series, yielding a statistical LOD_95_ (95 % detection probability) of 0.075 CFU/mL. While this modeled sensitivity reflects excellent analytical performance, it lies below the experimentally tested range; therefore, 100 CFU/mL was adopted as the validated LOD for clinical and quality-control use. Together, these results demonstrate that all three assays show high linearity, high reproducibility, and consistent extraction-method performance, including the rapid extraction-free D2P workflow.

**Table 1:** Percent positive detection at each input concentration (CFU/mL) for three extraction methods.

Concentration range	CFU/mL	n	KingFisher (% pos)	D2P (% pos)	Qiagen (% pos)
High (saturation)	1.0 × 10^7^	9	100	100	100
	1.0 × 10^6^	9	100	100	100
	1.0 × 10^5^	9	100	100	100
	1.0 × 10^4^	9	100	100	100
LOD region	1.0 × 10^3^	24	100	100	100
	1.0 × 10^2^	24	100	100	100
Sub-LOD	1.0 × 10^1^	24	75	70	60
	1.0 × 10^0^	24	20	30	35

*Note*: Empirical LOD for all methods was defined as the lowest concentration with ≥95 % positive replicates and confirmed as 100 CFU/mL.

These findings demonstrate that D2P provides diagnostic accuracy comparable to Qiagen, while eliminating labor-intensive extraction steps, making it a practical and efficient alternative for high-throughput and resource-limited settings. Although KingFisher offers automation benefits, its lower sensitivity and specificity suggest that D2P may be a superior choice for clinical laboratories requiring rapid and cost-effective pathogen detection.

### 3.2 Phase 2: comparative analysis of D2P and KingFisher in residual clinical isolates

In Phase 2, the performance of Direct-to-PCR (D2P) extraction-free processing was compared with KingFisher (magnetic bead-based extraction) for the detection of CTX-M Group 1 ESBL-producing pathogens in residual clinical urine specimens (Table 2). The analyzed microorganisms included *Escherichia coli*, *Klebsiella pneumoniae*, *Enterobacter cloacae*, *Klebsiella oxytoca*, *Klebsiella aerogenes*, *Citrobacter* spp., *Proteus mirabilis*, *Proteus vulgaris*, *Morganella morganii*, and *Serratia marcescens*. To comprehensively evaluate the effectiveness of both methods, key diagnostic performance metrics—sensitivity, specificity, accuracy, positive predictive value (PPV), and negative predictive value (NPV)—were assessed.

**Table 2:** Phase 2 comparative performance metrics (D2P method).

Microorganism	TP (D2P)	TN (D2P)	FP (D2P)	FN (D2P)	Sensitivity (D2P)	Specificity (D2P)	Accuracy (D2P)	PPV (D2P)	NPV (D2P)
*E. coli*	26	14	0	0	100.00 %	100.00 %	100.00 %	1.00	1.00
*K. pneumoniae*	14	25	0	1	93.33 %	100.00 %	100.00 %	1.00	0.96
*E. cloacae*	13	26	0	1	92.86 %	100.00 %	100.00 %	1.00	0.96
*K. oxytoca*	2	37	1	0	100.00 %	97.37 %	97.37 %	0.67	1.00
K. aerogenes	3	37	0	0	100.00 %	100.00 %	100.00 %	1.00	1.00
Citrobacter spp.	2	38	0	0	100.00 %	100.00 %	100.00 %	1.00	1.00
*P. mirabilis*	7	33	0	0	100.00 %	100.00 %	100.00 %	1.00	1.00
*P. vulgaris*	2	38	0	0	100.00 %	100.00 %	100.00 %	1.00	1.00
*M. morganii*	5	35	0	0	100.00 %	100.00 %	100.00 %	1.00	1.00
*S. marcescens*	0	40	0	0	NA	100.00 %	100.00 %	NA	1.00
CTX-M-Grp1	10	29	1	0	100.00 %	96.67 %	96.67 %	0.67	1.00

D2P demonstrated 100 % sensitivity for *E. coli*, *K. oxytoca*, *K. aerogenes*, *Citrobacter* spp., *P. mirabilis*, *P. vulgaris*, and *M. morganii*, confirming zero false negatives for these organisms. However, for *K. pneumoniae* and *E. cloacae*, D2P exhibited slightly lower sensitivity (93.33 % and 92.86 %), attributable to minor false negatives. Specificity remained 100 % across all organisms except *K. oxytoca* (97.37 %), which had one false positive. Accuracy values mirrored this trend, with D2P achieving 100 % accuracy for all tested organisms except *K. oxytoca* (97.37 %). Additionally, D2P exhibited consistently high PPV (≥0.96) and NPV (≥0.96), reinforcing its strong diagnostic reliability.

Both D2P and KingFisher achieved 100 % sensitivity for *E. coli* and *P. mirabilis*, demonstrating high nucleic acid detection efficiency for these organisms. However, for *K. pneumoniae* and *E. cloacae*, D2P demonstrated slightly superior sensitivity (93.33 % and 92.86 %, respectively), whereas KingFisher showed slight reductions due to false negatives. Specificity remained 100 % across all tested pathogens in both methods, reflecting minimal false positives and high target detection fidelity. D2P maintained 100 % accuracy for *E. coli*, *P. mirabilis*, *P. vulgaris*, and *M. morganii*, confirming reliable pathogen detection. However, *K. pneumoniae*, *E. cloacae*, and *K. oxytoca* showed minor reductions in accuracy with KingFisher, which was attributed to increased false negatives. D2P further exhibited robust PPV and NPV values (≥0.96), reaffirming its high diagnostic precision and predictive reliability.

These results establish that D2P provides diagnostic accuracy comparable to, or exceeding, KingFisher, particularly in detecting *E. coli* and Proteus species, while offering a streamlined, extraction-free workflow. While KingFisher benefits from automation, its slightly lower sensitivity and accuracy, particularly for *K. pneumoniae* and *E. cloacae*, suggest potential limitations in certain clinical applications. Given D2P's high diagnostic performance, elimination of labor-intensive extraction steps, and cost-effectiveness, it represents an efficient alternative for the detection of CTX-M Group 1 ESBL-producing Enterobacteriaceae in clinical settings.

## 4 Discussion

The findings of this study support the viability of extraction-free technology (D2P) as a practical, efficient, and scalable alternative to traditional nucleic acid extraction methods for detecting CTX-M Group 1 ESBL genes. As molecular diagnostics continue to play an essential role in infectious disease management, particularly in antimicrobial resistance (AMR) surveillance, the need for rapid, cost-effective, and high-throughput methodologies has become increasingly urgent. Resource-limited laboratories and those seeking to optimize workflow efficiencies stand to benefit significantly from D2P's ability to streamline nucleic acid processing, eliminate costly reagents, and reduce hands-on time. These advantages address some of the most pressing challenges faced by laboratories worldwide, particularly in decentralized healthcare settings where access to molecular diagnostics is often constrained [8].

Traditional nucleic acid extraction methods, such as silica column-based (Qiagen) and magnetic bead-based (KingFisher) extractions, are well-established but suffer from various limitations in resource-constrained settings. These techniques require specialized equipment, multiple processing steps, trained personnel, and a consistent supply of reagents, all of which present barriers in low-income countries, rural healthcare centers, and outbreak response laboratories [9]. The dependency on single-use consumables and proprietary reagents also creates supply chain vulnerabilities, a challenge that became acutely evident during the COVID-19 pandemic, where laboratories worldwide struggled with shortages of essential extraction kits [10]. In contrast, D2P eliminates the need for these costly and logistically demanding steps, allowing for direct nucleic acid amplification from clinical samples without prior extraction. This reduces operational costs, minimizes reliance on external supply chains, and facilitates point-of-care diagnostics in settings where traditional extraction methods are impractical.

Beyond its implications for resource-limited settings, D2P presents a compelling advantage for laboratories seeking to enhance diagnostic efficiency. Traditional nucleic acid extraction can take up to 120 min to process, creating workflow bottlenecks in high-throughput environments such as reference laboratories, hospital microbiology departments, and AMR surveillance programs [11]. Our study demonstrated that D2P reduces total processing time by over 60 %, making an important contribution to faster diagnostic turnaround (∼45 min) while maintaining high sensitivity, specificity, and accuracy. The removal of multiple manual handling steps also mitigates the risk of cross-contamination, a common concern in high-throughput diagnostic settings [12]. Moreover, the ability of D2P to integrate with automation further enhances its scalability, making it an attractive solution for laboratories that need to process large sample volumes efficiently while maintaining high diagnostic integrity.

The clinical implications of D2P-based extraction-free diagnostics extend beyond efficiency and cost-effectiveness. The rising prevalence of CTX-M Group 1-producing Enterobacteriaceae poses a significant challenge to antibiotic stewardship and infection control measures [13]. Rapid and accurate detection of these resistance genes is critical for guiding empiric antibiotic therapy and preventing the spread of multidrug-resistant pathogens. By enabling faster turnaround times, D2P has the potential to improve patient management, optimize antimicrobial prescribing, and reduce the burden on hospital microbiology services. Furthermore, in low-resource settings where AMR surveillance remains limited, the adoption of D2P-based diagnostics could help bridge diagnostic gaps, enabling real-time monitoring of resistance trends and more effective public health interventions.

### 4.1 Limitations and further research

Despite its numerous advantages, the adoption of D2P methodologies requires further validation across diverse clinical contexts. One of the primary challenges in extraction-free PCR is the presence of potential PCR inhibitors in unprocessed samples, which can interfere with amplification efficiency ([15],[14]). Although D2P uses optimized lysis buffers to mitigate these effects, future studies should explore additional strategies, such as the development of inhibitor-resistant enzymes and buffer formulations tailored to different sample types. Additionally, while extraction-free techniques have been widely studied in viral diagnostics—particularly for SARS-CoV-2 RNA detection—their application in bacterial pathogen detection, including CTX-M ESBL genes, remains underexplored. Given that CTX-M resistance determinants are primarily plasmid-encoded, further research is warranted to assess D2P's ability to detect plasmid-mediated resistance genes in polymicrobial infections and complex clinical matrices such as blood and stool samples.

## 5 Conclusion

The findings of this study demonstrate that D2P extraction-free technology is a scientifically viable alternative to conventional silica column-based and magnetic bead-based nucleic acid extraction methods. It offers a practical, scalable, and cost-effective solution, particularly for resource-limited and high-throughput clinical laboratories. By eliminating the need for proprietary reagents, reducing processing time, and integrating with automated platforms, D2P enhances workflow efficiency and accessibility. While further research is needed to optimize its application across diverse clinical sample types, the evidence presented here supports its potential to modify antimicrobial resistance gene detection, particularly for CTX-M ESBLs. As antibiotic resistance continues to escalate globally, the adoption of accessible, high-efficiency diagnostic solutions like D2P will be critical for strengthening infectious disease surveillance, guiding antimicrobial stewardship, and improving patient outcomes worldwide.

## CRediT authorship contribution statement

**Sadia Almas:** Validation, Investigation, Formal analysis. **Rob E. Carpenter:** Writing – review & editing, Writing – original draft, Conceptualization. **Vaibhav K. Tamrakar:** Validation, Investigation, Formal analysis. **Aditya Sharma:** Data curation. **Kamalpreet Suri:** Data curation. **Salima Karki:** Data curation. **Katelyn Kyser:** Data curation. **Randy Sronce:** Data curation. **Rahul Sharma:** Writing – review & editing, Validation, Supervision, Project administration, Methodology, Formal analysis, Conceptualization.

## Ethics approval and consent to participate

This study was conducted in accordance with institutional ethical guidelines and complied with all relevant regulatory and ethical standards for research involving microbial and clinical specimens. The use of de-identified residual clinical samples exempted the study from requiring Institutional Review Board (IRB) approval, as no direct patient involvement, identifiable information, or human subject interventions were included. All microbiological procedures were performed following biosafety and bioethics protocols to ensure compliance with good laboratory practices (GLP) and infection control standards.

## Funding sources

This research received no external funding.

## Declaration of competing interest

The authors declare the following financial interests: Rob E. Carpenter and Rahul Sharma have financial interests in Scienetix, LLC. This relationship has been disclosed in accordance with journal guidelines, and all efforts have been made to ensure the objectivity and integrity of the research presented in this study. The remaining authors declare no competing interests.

## Data Availability

Data will be made available on request.
